# The Umeå University Database of Facial Expressions: A Validation Study

**DOI:** 10.2196/jmir.2196

**Published:** 2012-10-09

**Authors:** Hanna Samuelsson, Karl Jarnvik, Hanna Henningsson, Josefin Andersson, Per Carlbring

**Affiliations:** ^1^Department of PsychologyUniversity of UmeåUmeåSweden

**Keywords:** Face, Expression, Stimulus set, Emotion, Multiracial, Validity, Reliability

## Abstract

**Background:**

A set of face stimuli, called the Umeå University Database of Facial Expressions, is described. The set consists of 30 female and 30 male models aged 17–67 years (M = 30.19, SD = 10.66). Each model shows seven different facial expressions (angry, surprised, happy, sad, neutral, afraid, and disgusted). Most models are ethnic Swedes but models of Central European, Arabic, and Asian origin are also included.

**Objective:**

Creating and validating a new database of facial expressions that can be used for scientific experiments.

**Methods:**

The images, presented in random order one at a time, were validated by 526 volunteers rating on average 125 images on seven 10-point Likert-type scales ranging from “completely disagree” to “completely agree” for each emotion.

**Results:**

The proportion of the aggregated results that were correctly classified was considered to be high (M = 88%).

**Conclusions:**

The results lend empirical support for the validity of this set of facial expressions. The set can be used freely by the scientific community.

## Introduction

There is a wealth of published research into face perception, face processing, and facial expressions using images of facial expressions of emotions [1]. Images of facial expressions of emotions are frequently used in research on emotions and are increasingly being used in neuroscience [2]. Recently, their use has been extended to the treatment of anxiety and depression, using a modified dot-probe task [3]. This paper presents the Umeå University Database of Facial Expressions, which is freely available to researchers. We present information about the database, along with the results of an Internet-based validation study.

The human face is an integral part of daily life. Facial muscles allow a wide range of expressions and functions [4]. These expressions communicate emotions in interaction with others and are an important part of the emotional experience [5]. The link between emotion and facial expression is the main driver of research interest in facial expressions and their interpretation [1].

Ekman and Friesen published their pioneering Pictures of Facial Affect (PFA) in 1976, which became the most frequently used set in research [2], greatly improving our understanding of the universality of the facial expression of emotion [6]. Today, however, this set of facial expressions has limitations, such as the small number of images, and is considered rather dated in regard to quality and the range of models, which adversely affects its ecological validity [2,7]. This and some other currently available sets do not contain models representing different ethnicities (KDEF, see [2,8]; FACES [9]).

The understanding and perception of emotions has been shown to be more accurate if those that are evaluating emotional expressions have the same ethnicity and national and regional background as the expressers. This may be because people of different ethnicities develop different nuances in their expressions. However, when different cultural groups spend more time together, the in-group advantage decreases [10]. Evaluation of facial expressions of emotions is also influenced by stereotypical ideas about people of other ethnicities [11].

The NimStim Set of Facial Expressions (NimStim) [1], the Japanese and Caucasian Facial Expressions of Emotion (JACFEE) [12], and the Montreal Set of Facial Displays of Emotion (MSFDE) [13] include models of different ethnicities but contain fewer than 145 images in total (JACFEE, MSFDE) [2]. They lack representation of different age groups, and it is not possible to use them in Internet-based experiments, even with password protection (NimStim) [1].

This project has attempted to address problems identified in previous sets of facial expressions and validation studies. The aim of producing the Umeå University Database of Facial Expressions was to create a database for Internet-based research, containing a large number of images across a spectrum of age, ethnicity, and gender.

This database has several advantages. First, it contains a large number of color images—a total of 424, posed by 60 models (2720x4080 pixels). The models express the most consistently recognized facial expressions of emotions, which are anger, surprise, happiness, sadness, fear, and disgust [14] as well as a neutral expression. Databases containing a large number of images are preferable because research often requires a large body of stimulus material in order to avoid the effects of habituation [2]. Second, the models represent different age groups, ethnicities, and genders, which gives the database good ecological validity with regard to these variables. Third, the database is available with password protection for scientific experiments on the Internet.

The aim of this validation study was to examine the extent to which facial expressions as depicted in the images were correctly interpreted as the intended emotion. It was done over the Internet in order to recruit participants with as great a range as possible of age, gender, and ethnicity. Swedish law does not, however, permit the registration of individual ethnicity. However, researchers based in a country without this restriction on reporting of individual ethnicities are free to do so after inspection of the photographs.

The genders of both model and rater may influence evaluation of facial expressions [15-19]. The age of the rater also plays an important role in the correct recognition of facial expression [20,21]. Validation studies commonly include only university or college students [2,7,8,12]. In order to reach a more heterogeneous group of people, we recruited participants both within and outside higher education.

Each image was evaluated by participants. Nuanced answer options were used in the validation study in order to reduce the risk of influencing responses to a specific expression. Response scales with fixed response options can be problematic, as different response scale formats may influence the results obtained [22]. Predetermined emotion labels can be regarded as a contextual variable that influences the participant’s response to a specific expression [22,23]. In this validation study, participants were therefore given the option of rating expressions for several different emotions.

We hypothesized that the Internet-based validation study would provide sufficient data to support the validity of the Umeå University Database of Facial Expressions.

## Methods

### Participants

Data were collected from 526 participants. The mean age was 37.7 years (18–73, SD =13.0). 70% (369/526) were female and 30% (157/526) were male. Participants were recruited by disseminating information about the study via the local Swedish newspaper. All those who volunteered were allowed to take part in the study, and no financial compensation or remuneration was given.

### Stimuli

The stimuli were 424 facial images from the Umeå University Database of Facial Expressions. A total of 60 subjects participated as amateur models (30 female, 30 male; 17-67 years old; M=30.19, SD=10.66). Most of these models were ethnic Swedes, but models of Central European, Arabic, and Asian origin were also included. During the photography session, models were instructed to display seven different facial expressions (angry, surprised, happy, sad, neutral, afraid, and disgusted). Instructions on how to make the facial expressions were based on the work of Ekman [24] and Ekman and Friesen [6] and presented to models before and during the sessions. Before the shoots, models were encouraged to practice making the facial expressions, and during the shoots models were instructed to make the expressions as they saw fit, to look at pictures of facial expressions (POFA) [25] and to move certain muscle groups. Models were instructed not to wear make-up. The shoots took place at Umeå University. Models were compensated for their participation by receiving uncompressed high-quality personal photographs. They also signed a legal agreement allowing the images to be used in research and education.

### Selection of Images

The photo shoots produced over 8,000 images. The best image of each expression from each model was chosen to be validated empirically. However, a clear decision could not be made in four instances, and these images were therefore added to the validation phase, making a total of 424.

### Validation Procedure

The validation procedure took place on the Internet. Before obtaining access to the images, the potential participant had to register his/her age, gender, and email address. A confirmatory email, including a unique login link, was sent to the registered email address, ensuring that all participants had registered a valid email address. Instructions to participants were to sit alone in a quiet, private setting and base the evaluations on their own opinion. Participants evaluated the images at their own pace and were free to evaluate as many images as they wished. They were allowed to discontinue the evaluation at any time and were free to return and continue at another time during a two-week period in October 2011. Images were randomly presented to each participant. However, each of the 424 images was presented only once. 526 participants started the validation process, rating an average of 125.5 out of 424 faces (SD=137.4).

**Figure 1:**
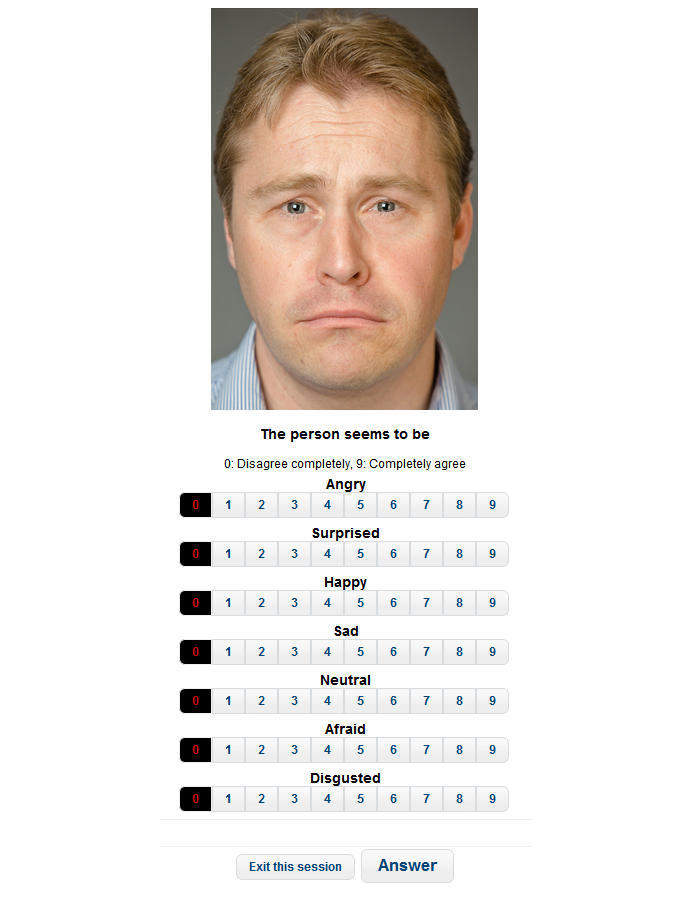
A screen shot of the web-based validation.

Each of the 424 images (320x480 pixels, color) was presented on its own with the text “This person seems to be…” above each image. As shown in Figure 1, the options “angry”, “surprised”, “happy”, “sad”, “neutral”, “afraid”, and “disgusted” were presented below each image together with a 10-point Likert-type scale ranging from “completely disagree” to “completely agree”. Participants could specify to what extent they agreed or disagreed with one or more of the listed emotions.

### Data Analytic Procedure

We used a binary logistic model (specified through generalized linear equations), and variance–covariance for all models was assumed to be block diagonal but independent within a block defined by individual, which implies that we assumed that the scoring of one image did not affect the score given by that individual to the next randomized image.

The seven outcome variables were defined as 1/0 for each “true” emotion. The independent factors were gender and age of the rater and model and the rating score for the seven emotions. We studied the adjusted association between each outcome and the 11 independent factors. We present the estimated odds ratios and their 95% Wald confidence intervals (CIs) and their significance (see Supplemental Tables 1–7 in Appendix 1). All tests were two-sided. The results were considered significant if *P*<.05. All analyses were performed using SPSS, version 20 (SPSS, Inc., Chicago, IL).

We considered an image to be correctly classified if the highest score was given to the emotion corresponding to the true emotion. For example, if the emotion “sad” was scored 7 and the other emotions between 0 and 6 points, then sad would be counted as the response. That response would then be compared to the intended emotion when calculating the hit rate. In addition, in order to obtain a measure of the reliability of the interpretation, we also calculated the sum of the scores given to emotions that did not correspond to the true emotion, and the number of emotions rated.

Table 1 describes the scores given by raters for all images where models attempted to display a given emotion in the following columns: 1, “Percentage correctly perceived”, how often the emotion that models displayed was rated higher than all other emotions; 2, “Number of unintended emotions scored (0−6)”, how many unintended emotions were given a score of greater than 0; and 3, “Total score (0−9) given to unintended emotions”, the sum of scores given to emotions that did not correspond to the true emotion.

## Results

### Validity

The validity measure (proportion interpreted correctly) was performed for every image. The data for these 424 individual images are presented separately on the Internet database. The proportion correctly interpreted for each portrayed emotion is, however, shown in Table 1. The overall value for the aggregated sum of results was considered high (mean=88%). Five out of the seven expressions had a mean percentage of correct interpretation of over 90%, while the emotions of fear and sadness had a mean percentage of 73% and 78% respectively. Ratings for emotions other than that intended are considered to be low (mean 0.13–0.65). There was a difference between the emotions in ratings given to emotions other than those intended (mean range from 0.38 points for happiness to 3.15 points for fear).

As shown in Table 2, levels of incorrect perception of expressed emotions were, with a few exceptions (eg, the intended facial expression of fear being perceived as surprise), consistently low.

**Table 1:** Summary of the proportion of images correctly perceived, number of unintended emotions scored and the total scores given to unintended emotions.

Emotion expressed	Number of images	Proportion correctly perceived (%)			Number of unintended emotions scored (0–6)			Total score given to unintended emotions (0–9)		
		Mean^a^	Min^b^	Max^c^	Mean^a^	Min^b^	Max^c^	Mean^a^	Min^b^	Max^c^
Anger (n=9581)	61	94	72	100	0.25	0.07	0.73	0.87	0.12	2.57
Surprise (n=9357)	60	94	76	99	0.33	0.14	0.66	1.25	0.42	3.26
Happiness (n=9721)	62	98	85	100	0.13	0.05	0.44	0.38	0.08	1.65
Sadness (n=9393)	61	78	25	98	0.54	0.14	1.23	2.41	0.45	6.55
Neutral (n=9406)	60	91	56	99	0.38	0.14	0.94	1.21	0.36	4.21
Fear (n=9211)	60	73	39	95	0.65	0.33	1.08	3.15	1.43	6.32
Disgust (n=9325)	60	90	60	100	0.36	0.10	0.86	1.42	0.22	4.23
Total (n=65994)	424	88	25	100	0.38	0.05	1.23	1.52	0.08	6.55

Note: An image was considered to be correctly classified if the highest score was given to the emotion corresponding to the true emotion.

^a ^Mean proportion of correct perception (n=9211–9721).

^b ^The value for the image with the lowest proportion of correct perception.

^c ^The value for the image with the highest proportion of correct perception.

**Table 2:** Confusion matrix for images of expressed emotion and rater response (only scores 7–9 shown).

Expressed emotion	Rater response (7–9) (%)						
	Anger	Surprise	Happiness	Sadness	Neutral	Fear	Disgust
Anger	74.6^a^	0.4	0.2	0.9	0.5	0.8	0.9
Surprise	0.2	81.7^a^	1.0	0.2	0.5	3.7	0.3
Happiness	0.2	0.3	92.5^a^	0.3	0.5	0.2	0.2
Sadness	1.1	1.0	0.4	55.6^a^	5.9	3.9	2.3
Neutral	1.0	0.8	0.4	1.7	81.6^a^	0.6	0.1
Fear	2.6	14.2	0.6	0.9	0.5	55.5^a^	1.7
Disgust	2.2	0.9	0.3	2.5	0.2	0.8	71.8^a^

^a ^Intended emotion.

### Odds Ratio

The odds ratios (presented in the supplemental tables in Appendix 1), ie, the relation between ratings 9 and 0, are high. The highest odds ratio was found in the expression of happiness (OR=1945.6, *P<.*0001), and the lowest was in fear (OR=72.0, *P*=*<.*0001).

The most noteworthy results relating to the four background variables (model age, model gender, rater age, and rater gender), presented in the supplemental tables in Appendix 1, were in the expressions of surprise and anger. Female facial models aged ≥46 years (OR=0.4, *P*<.05) and 26–45 years (OR=0.8, *P<*.05) were significantly less strongly associated with the expression anger in comparison with the reference group, but at the same time, significantly more strongly associated with the expression of surprise (OR=1.8, and OR=1.1, *P<*.05).

Female facial models were more frequently significantly associated with three of the intended expressions in comparison with male facial models. Those were the expressions anger (OR=1.2, *P*<.05), surprise (OR=1.2, *P*<.05) and neutral (OR=1.9, *P*<.05). There were no statistically significant differences in the expression of happiness, fear, and disgust. The expression of sadness was more frequently associated with male models than female models (OR=0.6, *P*<.05).

## Discussion

### Principal Results

The purpose of this study was to present a database of facial expressions and the results of an Internet-based validation study. The database contains 424 color images of models across a spectrum of age, ethnicity, and gender expressing a variety of different emotions. The database is freely available for scientific experiments both online and offline.

The validity of the database was based on how accurate the raters were in identifying the expressions in the presented images. Scores were generally high. The overall mean proportion of this database that was correctly interpreted was 88%. The corresponding values are 79% for NimStim [1] and 88% for Pictures of Facial Affect [25], with the Karolinska Directed Emotional Faces achieving a mean biased hit rate of 72% [2].

The results did not show any consistent advantage related to age or gender in either the models or in the validating participants. There were significant differences when the seven expressions were studied individually, but the stronger and weaker association varies across the four background variables. Hall and Matsumoto found that women made more correct interpretations than men when multiple scales were used [26]. These results were not replicated in our study, with the exception that women were better than men at identifying the neutral emotion and worse than men at recognizing disgust.

The results of previous studies [27] of the effect of age and gender have suggested that even higher identification scores are obtained if images are validated exclusively by women aged 20-30 years. However, our data did not show any consistently significant differences between gender groups or age groups, and previous results were not replicated in this study population. The only significant differences were that raters aged ≥46 years were better at identifying disgust and worse at identifying anger and that raters aged 26-45 were worse at identifying happiness than the reference group. That lower capacity of older raters to correctly identify anger is consistent with the results of Ebner et al [9].

The results of our study show that facial expressions of people ≥46 years showing anger, fear, and sadness were less reliably identified than those posed by younger faces aged ≤25 years. Faces of participants aged 26-45 years portraying anger, neutral, and disgust were also less reliably identified than the same expressions in younger people aged ≤25 years. This is consistent with the findings of Ebner et al [9] who showed that angry, disgusted, happy, neutral, and sad expressions were less accurately identified in older faces than in the faces of the young or middle-aged. Disgusted, neutral, and sad middle-aged faces were less accurately identified than young faces portraying the same emotions.

The validation study was Internet-based. A large number of participants from different age groups evaluated the images, which provides this study with a more heterogeneous population of raters than previous studies [2,7,8,12]. The number of ratings for each image was higher than in previous studies [1,2,8]. While this type of validation has merits, we had no control over how the raters were complying with the task or if they instead carried out the validation in a detrimental way.

As response scales with fixed response options can be problematic, Russell [22] recommended more studies with quantitative ratings on multiple scales. These are considered more neutral as they are not biased towards a single expression. Therefore, we chose a ten-degree Likert-type scale ranging from “completely disagree” to “completely agree”. Participants could choose to agree on one or more of the response scales. Giving the participants the opportunity to rate each image on a continuum and to choose to rate several emotions for each image provided important information about each image, enabling an assessment of how reliably it was depicting the intended emotion as compared to other emotions.

Some facial expressions of emotion are easier to identify correctly than others. In validation studies, happy facial expressions are usually recognized more reliably than negative facial expressions [1,2,25,28,29]. In this validation study, happy was the facial expression that had the highest proportion of correct identification and the lowest association with other emotions. These results are consistent with previous findings that happiness is the facial expression that is the most reliably identified and the least likely to be confused with other facial expressions [12].

Sadness and fear had the lowest proportion of correct identification, also consistent with previous research [2,12]. These expressions were also confused with other expressions to a greater extent. In particular, raters often thought that fear had an element of surprise. This confusion may be due to the similarities in these two facial expressions, with the eyes being wide open in both. It may be difficult for untrained models to make the facial movements necessary to distinguish these expressions. There may also be a measure of confusion on the basis of interpretation, because when fear is experienced, it is often preceded by surprise [24].

The method of creating facial expressions can affect their interpretation. Currently existing databases have been produced by instructing the photo shoot models in two different ways. One is to instruct the models to move particular muscle groups while making the facial expressions [8,25], and the other is to instruct them to make the emotional expressions as they see fit [1].

One advantage of asking models to move particular muscle groups is that it creates uniform expressions. The disadvantage is that the ecological validity may be affected [22]. Naturally produced facial expressions can be perceived as more authentic, but the variation within the same expression may be greater [22], and this could be regarded as a background variable in scientific experiments [1]. When models follow instructions about which muscles to move or imitate a picture, a larger proportion of expressions are correctly identified compared to studies in which the models made the expressions as they saw fit or spontaneous expressions were induced [10].

As we wanted models to make authentic expressions and still maintain uniformity within the same emotional expressions, the instructions given to models were a combination of the instructions used in previous studies. The models in the Umeå University Database of Facial Expressions were instructed to make the expressions as they saw fit, to look at pictures of facial expressions, and to move certain muscle groups. 

### Limitations

The database has, however, a number of shortcomings. First, as the validation study was Internet-based, it was difficult to control for the authenticity of participant responses and other contextual variables, eg, how closely participants followed the instructions. However, the requirement for personal information such as name, age, gender, and email address should have decreased the risk of non-valid answers. In addition, the relatively large number of participants (n=526) would have reduced the impact of deliberately false responses. The lack of remuneration also meant there was no financial reward in providing false responses.

Second, models may have validated their own images, which may have inflated the proportion of correct identification in the database. However, the number of models who may have validated their own images was small in relation to the large number of ratings made for each image.

Third, there may have been a subjective interpretation of the meaning of the response scales. The scale steps between 0 and 9 could have been interpreted as a measure of intensity, authenticity, or purity. However, giving the participants the opportunity to rate every image on a continuum and to rate for several expressions, provided important information about each image. Valuable information about the extent to which each image was rated for expressions other than the one intended is available online, as well as the proportion correctly identified for each image.

A fourth limitation is that a forced-choice scale was used to calculate the proportion of correct identification. The response scale that received the highest score was regarded as the respondent’s answer. And since there was no “none of the above” option included, this has probably resulted in a higher proportion of correct identification than if this option had been included.

A fifth weakness is that no member of the research team instructing the models during the photo shoots, and selecting images for validation was certified according to the Facial Action Coding System (FACS) [30]. FACS is a guide to the categorization of facial movements according to the muscles used in producing them. But this would not have been entirely satisfactory either, as using FACS to create images has resulted in American “dialects” of facial expressions [10]. Not using FACS could be regarded as an advantage when the images are to be used with untrained participants.

Finally, the instruction not to wear make-up was not followed by all participants, which may bias the interpretation of the images. However, the resulting images may more closely resemble the facial expressions seen in real life.

### Conclusion

The goal of creating the Umeå University Database of Facial Expressions was to provide the scientific community with an online database for scientific experiments. The database consists of a large and contemporary set of images showing models across a spectrum of age, ethnicity, and gender. The Internet-based validity study obtained a larger number of ratings for each image compared to previous validation studies, and it has a higher proportion of correct identification compared to many existing databases. However, the validity of the Umeå University Database of Facial Expressions needs to be tested by further validation studies of similar or different design. Finally, we invite the scientific community to help expand the database by allowing inclusion of additional models to provide a more representative sample of populations. Obviously any added faces would first need to be validated to ensure high standards.

## Multimedia Appendix 1

Supplemental Tables 1-7. Factors associated with images.
