# Supplementary material for: The Umeå University Database of Facial Expressions: A Validation Study
Source: J Med Internet Res. 2012 Oct 9;14(5):e136. doi: 10.2196/jmir.2196 (PMC3510711; doi:10.2196/jmir.2196)
Supplement: Supplementary file 1 [file jmir_v14i5e136_app1.pdf]

Supplemental Table 1. *Factors associated with images portraying anger. The total falls short of 100% because some of the responses made at each step of the response rating scale for anger were actually made for portrayals of other, unintended emotions, and these figures appear in the corresponding tables for each emotion.*

	Rating	Proportion	Ratings made for images portraying anger (n=9581)	Ratings made for all images	Ratings made for portrayals of anger as a proportion of all ratings #	Adjusted odds ratio	*P*	95 % Wald CI	
**Anger (n=9581)**	9	46.0%	4403	4702	93.6%	213.6	.00	164.6	277.1
	7–8	28.7%	2746	3135	87.6%	137.9	.00	107.8	176.3
	3–6	19.7%	1886	3575	52.8%	38.0	.00	29.9	48.4
	1–2	1.8%	176	1823	9.7%	8.6	.00	6.1	12.2
	0	3.9%	370	52759	0.7%	1.0	ref.	ref.	ref.
**Surprise**	9	0.2%	15	5543	0.3%	0.0	.00	0.0	0.0
	7–8	0.3%	28	3728	0.8%	0.1	.00	0.0	0.1
	3–6	1.3%	120	3446	3.5%	0.3	.00	0.2	0.4
	1–2	1.8%	175	1557	11.2%	0.8	.13	0.6	1.1
	0	96.5%	9243	51720	17.9%	1.0	ref.	ref.	ref.
**Happiness**	9	0.1%	10	7287	0.1%	0.0	.00	0.0	0.0
	7–8	0.1%	8	1968	0.4%	0.0	.00	0.0	0.1
	3–6	0.5%	48	1315	3.7%	0.3	.00	0.2	0.5
	1–2	1.6%	149	1241	12.0%	1.2	.30	0.9	1.6
	0	97.8%	9366	54183	17.3%	1.0	ref.	ref.	ref.
**Sadness**	9	0.3%	24	3004	0.8%	0.1	.00	0.0	0.1
	7–8	0.6%	59	2819	2.1%	0.1	.00	0.1	0.2
	3–6	2.6%	252	3602	7.0%	0.3	.00	0.2	0.4
	1–2	2.3%	225	1803	12.5%	0.8	.09	0.6	1.0
	0	94.2%	9021	54766	16.5%	1.0	ref.	ref.	ref.
**Neutral**	9	0.2%	17	5802	0.3%	0.0	.00	0.0	0.0
	7–8	0.3%	30	2640	1.1%	0.0	.00	0.0	0.1
	3–6	1.0%	97	1782	5.4%	0.1	.00	0.1	0.2
	1–2	1.4%	133	836	15.9%	0.9	.50	0.6	1.3
	0	97.1%	9304	54934	16.9%	1.0	ref.	ref.	ref.
**Fear**	9	0.2%	21	2877	0.7%	0.1	.00	0.0	0.1
	7–8	0.6%	56	3171	1.8%	0.2	.00	0.1	0.2
	3–6	2.0%	195	4083	4.8%	0.3	.00	0.2	0.4
	1–2	2.3%	222	2002	11.1%	0.8	.12	0.6	1.1
	0	94.8%	9087	53861	16.9%	1.0	ref.	ref.	ref.
**Disgust**	9	0.3%	31	4233	0.7%	0.1	.00	0.0	0.1
	7–8	0.6%	60	2986	2.0%	0.1	.00	0.0	0.1
	3–6	2.3%	217	3025	7.2%	0.2	.00	0.1	0.2

	1–2	2.3%	219	1297	16.9%	0.6	.00	0.5	0.9
	0	94.5%	9054	54453	16.6%	1.0	ref.	ref.	ref.
**Model age**	≥ 46	9.9%	952	6515	14.6%	0.4	.00	0.4	0.5
	26–45	53.9%	5166	36103	14.3%	0.8	.00	0.7	0.9
	≤ 25	36.1%	3463	23376	14.8%	1.0	ref.	ref.	ref.
**Model gender**	Female	49.1%	4707	33042	14.2%	1.2	.00	1.1	1.3
	Male	50.9%	4874	32952	14.8%	1.0	ref.	ref.	ref.
**Rater age**	≥ 46	35.3%	3385	23405	14.5%	0.8	.04	0.6	1.0
	26–45	50.5%	4840	33054	14.6%	0.9	.16	0.7	1.1
	≤ 25	14.2%	1356	9535	14.2%	1.0	ref.	ref.	ref.
**Rater gender**	Female	70.2%	6727	46600	14.4%	1.0	.85	0.9	1.2
	Male	29.8%	2854	19394	14.7%	1.0	ref.	ref.	ref.

Supplemental Table 2. *Factors associated with images portraying surprise. The total falls short of 100% because some of the responses made at each step of the response rating scale for surprise were actually made for portrayals of other, unintended emotions, and these figures appear in the corresponding tables for each emotion.*

	Rating	Proportion	Ratings made for images portraying surprise (n=9357)	Ratings made for all images	Ratings made for portrayals of surprise as a proportion of all ratings	Adjusted odds ratio	***P***	95 % Wald CI	
**Anger**	9	0.1%	9	4702	0.2%	0.0	.00	0.0	0.0
	7–8	0.1%	13	3135	0.4%	0.0	.00	0.0	0.1
	3–6	0.5%	44	3575	1.2%	0.1	.00	0.1	0.1
	1–2	1.2%	114	1823	6.3%	0.6	.01	0.4	0.9
	0	98.1%	9177	52759	17.4%	1.0	ref.	ref.	ref.
**Surprise (n=9357)**	9	52.5%	4909	5543	88.6%	112.6	.00	84.7	149.6
	7–8	29.3%	2739	3728	73.5%	53.5	.00	41.1	69.7
	3–6	12.8%	1198	3446	34.8%	17.7	.00	13,7	22,7
	1–2	1.3%	124	1557	8.0%	6.0	.00	4.4	8.1
	0	4.1%	387	51720	0.7%	1.0	ref.	ref.	ref.
**Happiness**	9	0.3%	26	7287	0.4%	0.0	.00	0.0	0.0
	7–8	0.7%	67	1968	3.4%	0.2	.00	0.1	0.3
	3–6	4.2%	389	1315	29.6%	1.0	.78	0.8	1.2
	1–2	3.8%	358	1241	28.8%	1.9	.00	1.3	2.9
	0	91.0%	8517	54183	15.7%	1.0	ref.	ref.	ref.
**Sadness**	9	0.0%	3	3004	0.1%	0.0	.00	0.0	0.0
	7–8	0.1%	12	2819	0.4%	0.0	.00	0.0	0.1
	3–6	0.5%	47	3602	1.3%	0.1	.00	0.1	0.2
	1–2	1.1%	106	1803	5.9%	0.6	.01	0.4	0.9
	0	98.2%	9189	54766	16.8%	1.0	ref.	ref.	ref.
**Neutral**	9	0.2%	23	5802	0.4%	0.0	.00	0.0	0.1
	7–8	0.3%	26	2640	1.0%	0.1	.00	0.1	0.1
	3–6	0.9%	82	1782	4.6%	0.3	.00	0.2	0.4
	1–2	1.2%	112	836	13.4%	2.3	.00	1.3	3.9
	0	97.4%	9114	54934	16.6%	1.0	ref.	ref.	ref.
**Fear**	9	1.2%	114	2877	4.0%	0.2	.00	0.1	0.2
	7–8	2.4%	228	3171	7.2%	0.2	.00	0.1	0.2
	3–6	7.1%	666	4083	16.3%	0.3	.00	0.2	0.3
	1–2	4.8%	452	2002	22.6%	0.5	.00	0.4	0.7
	0	84.4%	7897	53861	14.7%	1.0	ref.	ref.	ref.
**Disgust**	9	0.1%	14	4233	0.3%	0.0	.00	0.0	0.1
	7–8	0.1%	11	2986	0.4%	0.0	.00		
	3–6	0.6%	60	3025	2.0%	0.1	.00	0.1	0.1

	1–2	1.3%	122	1297	9.4%	0.4	.00	0.2	0.6
	0	97.8%	9150	54453	16.8%	1.0	ref.	ref.	ref.
**Model age**	≥ 46	10.2%	950	6515	14.6%	1.8	.00	1.6	2.1
	26–45	54.3%	5081	36103	14.1%	1.1	.01	1.0	1.2
	≤ 25	35.5%	3326	23376	14.2%	1.0	ref.	ref.	ref.
**Model gender**	Female	49.7%	4646	33042	14.1%	1.2	.00	1.1	1.3
	Male	50.3%	4711	32952	14.3%	1.0	ref.	ref.	ref.
**Rater age**	≥ 46	35.5%	3322	23405	14.2%	0.9	.41	0.7	1.1
	26–45	50.1%	4687	33054	14.2%	1.0	.86	0.8	1.2
	≤ 25	14.4%	1348	9535	14.1%	1.0	ref.	ref.	ref.
**Rater gender**	Female	70.6%	6604	46600	14.2%	0.9	.22	0.7	1.1
	Male	29.4%	2753	19394	14.2%	1.0	ref.	ref.	ref.

Supplemental Table 3. *Factors associated with images portraying happiness. The total falls short of 100% because some of the responses made at each step of the response rating scale for happiness were actually made for portrayals of other, unintended emotions, and these figures appear in the corresponding tables for each emotion.*

	Rating	Proportion	Ratings made for images portraying happiness (n=9721)	Ratings made for all images	Ratings made for portrayals of happiness as a proportion of all ratings	Adjusted odds ratio	*P*	95 % Wald CI	
**Anger**	9	0.1%	9	4702	0.2%	0.0	.00	0.0	0.1
	7–8	0.1%	7	3135	0.2%	0.0	.00	0.0	0.1
	3–6	0.4%	36	3575	1.0%	0.1	.00	0.1	0.2
	1–2	1.3%	123	1823	6.7%	0.6	.03	0.3	0.9
	0	98.2%	9546	52759	18.1%	1.0	ref.	ref.	ref.
**Surprise**	9	0.2%	17	5543	0.3%	0.0	.00	0.0	0.0
	7–8	0.2%	17	3728	0.5%	0.0	.00	0.0	0.0
	3–6	0.7%	68	3446	2.0%	0.1	.00	0.0	0.1
	1–2	1.4%	140	1557	9.0%	0.4	.00	0.3	0.7
	0	97.5%	9479	51720	18.3%	1.0	ref.	ref.	ref.
**Happiness (n=9721)**	9	73.9%	7186	7287	98.6%	1945.6	.00	1321.6	2864.4
	7–8	18.6%	1807	1968	91.8%	469.4	.00	319.9	688.6
	3–6	5.2%	504	1315	38.3%	66.5	.00	44.5	99.4
	1–2	0.3%	29	1241	2.3%	6.7	.00	4.1	11.2
	0	2.0%	195	54183	0.4%	1.0	ref.	ref.	ref.
**Sadness**	9	0.1%	14	3004	0.5%	0.1	.00	0.1	0.2
	7–8	0.2%	16	2819	0.6%	0.1	.00	0.1	0.2
	3–6	0.4%	35	3602	1.0%	0.3	.00	0.2	0.4
	1–2	1.3%	123	1803	6.8%	0.8	.43	0.5	1.4
	0	98.1%	9533	54766	17.4%	1.0	ref.	ref.	ref.
**Neutral**	9	0.2%	22	5802	0.4%	0.0	.00	0.0	0.1
	7–8	0.2%	22	2640	0.8%	0.1	.00	0.1	0.2
	3–6	1.0%	95	1782	5.3%	0.3	.00	0.2	0.5
	1–2	1.2%	121	836	14.5%	1.2	.38	0.8	1.9
	0	97.3%	9461	54934	17.2%	1.0	ref.	ref.	ref.
**Fear**	9	0.1%	9	2877	0.3%	0.1	.00	0.0	0.2
	7–8	0.1%	11	3171	0.3%	0.1	.00	0.0	0.2
	3–6	0.7%	69	4083	1.7%	0.3	.00	0.2	0.5
	1–2	1.6%	152	2002	7.6%	1.2	.45	0.8	1.7
	0	97.5%	9480	53861	17.6%	1.0	ref.	ref.	ref.
**Disgust**	9	0.1%	12	4233	0.3%	0.1	.00	0.0	0.1
	7–8	0.1%	11	2986	0.4%	0.1	.00	0.0	0.1
	3–6	0.2%	21	3025	0.7%	0.1	.00	0.1	0.2
	1–2	1.0%	95	1297	7.3%	0.6	.06	0.4	1.0

	0	98.6%	9582	54453	17.6%	1.0	ref.	ref.	ref.
**Model age**	≥ 46	9.6%	938	6515	14.4%	1.1	.38	0.9	1.5
	26–45	55.1%	5359	36103	14.8%	1.0	.96	0.9	1.2
	≤ 25	35.2%	3424	23376	14.6%	1.0	ref.	ref.	ref.
**Model gender**	Female	51.9%	5041	33042	15.3%	0.9	.42	0.8	1.1
	Male	48.1%	4680	32952	14.2%	1.0	ref.	ref.	ref.
**Rater age**	≥ 46	35.0%	3405	23405	14.5%	0.8	.20	0.6	1.1
	26–45	50.4%	4902	33054	14.8%	0.8	.04	0.6	1.0
	≤ 25	14.5%	1414	9535	14.8%	1.0	ref.	ref.	ref.
**Rater gender**	Female	70.6%	6866	46600	14.7%	0.9	.46	0.7	1.2
	Male	29.4%	2855	19394	14.7%	1.0	ref.	ref.	ref.

Supplemental Table 4. *Factors associated with images portraying sadness. The total falls short of 100% because some of the responses made at each step of the response rating scale for sadness were actually made for portrayals of other, unintended emotions, and these figures appear in the corresponding tables for each emotion.*

	Rating	Proportion	Ratings made for images portraying sadness (n=9393)	Ratings made for all images	Ratings made for portrayals of sadness as a proportion of all ratings	Adjusted odds ratio	*p*	95 % Wald CI	
**Anger**	9	0.4%	40	4702	0.9%	0.0	.00	0.0	0.1
	7–8	0.6%	59	3135	1.9%	0.1	.00	0.0	0.1
	3–6	3.8%	353	3575	9.9%	0.3	.00	0.2	0.4
	1–2	3.4%	316	1823	17.3%	0.7	.00	0.6	0.8
	0	91.8%	8625	52759	16.3%	1.0	ref.	ref.	ref.
**Surprise**	9	0.4%	39	5543	0.7%	0.0	.00	0.0	0.1
	7–8	0.5%	51	3728	1.4%	0.1	.00	0.1	0.1
	3–6	2.6%	240	3446	7.0%	0.4	.00	0.3	0.5
	1–2	2.4%	221	1557	14.2%	0.8	.10	0.7	1.0
	0	94.1%	8842	51720	17.1%	1.0	ref.	ref.	ref.
**Happiness**	9	0.2%	17	7287	0.2%	0.0	.00	0.0	0.0
	7–8	0.2%	21	1968	1.1%	0.1	.00	0.0	0.1
	3–6	0.4%	36	1315	2.7%	0.2	.00	0.1	0.3
	1–2	1.3%	121	1241	9.8%	0.6	.00	0.4	0.8
	0	97.9%	9198	54183	17.0%	1.0	ref.	ref.	ref.
**Sadness (n=9393)**	9	29.8%	2796	3004	93.1%	97.1	.00	76.8	122.9
	7–8	25.9%	2430	2819	86.2%	55.5	.00	42.1	73.0
	3–6	24.1%	2262	3602	62.8%	19.3	.00	16.1	23.0
	1–2	4.0%	372	1803	20.6%	5.2	.00	4.2	6.6
	0	16.3%	1533	54766	2.8%	1.0	ref.	ref.	ref.
**Neutral**	9	2.7%	251	5802	4.3%	0.2	.00	0.2	0.2
	7–8	3.3%	307	2640	11.6%	0.3	.00	0.3	0.4
	3–6	4.7%	440	1782	24.7%	0.7	.01	0.6	0.9
	1–2	2.2%	207	836	24.8%	1.3	.12	0.9	1.8
	0	87.2%	8188	54934	14.9%	1.0	ref.	ref.	ref.
**Fear**	9	1.3%	125	2877	4.3%	0.2	.00	0.1	0.3
	7–8	2.6%	241	3171	7.6%	0.3	.00	0.2	0.4
	3–6	6.6%	623	4083	15.3%	0.6	.00	0.5	0.7
	1–2	4.1%	389	2002	19.4%	0.9	.32	0.7	1.1
	0	85.3%	8015	53861	14.9%	1.0	ref.	ref.	ref.
**Disgust**	9	0.9%	88	4233	2.1%	0.1	.00	0.1	0.1
	7–8	1.3%	126	2986	4.2%	0.2	.00	0.1	0.2
	3–6	4.9%	459	3025	15.2%	0.5	.00	0.4	0.6
	1–2	3.2%	301	1297	23.2%	1.2	.04	1.0	1.5
	0	89.6%	8419	54453	15.5%	1.0	ref.	ref.	ref.

**Model age**	≥ 46	9.9%	929	6515	14.3%	0.8	.01	0.7	0.9
	26–45	54.1%	5077	36103	14.1%	1.3	.00	1.2	1.4
	≤ 25	36.1%	3387	23376	14.5%	1.0	ref.	ref.	ref.
**Model gender**	Female	49.3%	4628	33042	14.0%	0.6	.00	0.5	0.6
	Male	50.7%	4765	32952	14.5%	1.0	ref.	ref.	ref.
**Rater age**	≥ 46	35.5%	3336	23405	14.3%	0.9	.24	0.7	1.1
	26–45	49.9%	4686	33054	14.2%	1.0	.60	0.8	1.1
	≤ 25	14.6%	1371	9535	14.4%	1.0	ref.	ref.	ref.
**Rater gender**	Female	70.8%	6651	46600	14.3%	1.1	.42	0.9	1.3
	Male	29.2%	2742	19394	14.1%	1.0	ref.	ref.	ref.

Supplemental Table 5. *Factors associated with images portraying neutral emotion. The total falls short of 100% because some of the responses made at each step of the response rating scale for neutral were actually made for portrayals of other, unintended emotions, and these figures appear in the corresponding tables for each emotion.*

	Rating	Proportion	Ratings made for images portraying neutral emotion (n=9406)	Ratings made for all images	Ratings made for portrayals of neutral emotion as a proportion of all ratings	Adjusted odds ratio	*P*	95 % Wald CI	
**Anger**	9	0.4%	33	4702	0.7%	0.1	.00	.07	.17
	7–8	0.7%	65	3135	2.1%	0.3	.00	.19	.41
	3–6	4.2%	391	3575	10.9%	0.9	.69	.73	1.23
	1–2	5.6%	531	1823	29.1%	1.7	.00	1.20	2.28
	0	89.2%	8386	52759	15.9%	1.0	ref.	ref.	ref.
**Surprise**	9	0.2%	21	5543	0.4%	0.1	.00	.03	.09
	7–8	0.5%	51	3728	1.4%	0.2	.00	.13	.27
	3–6	1.5%	145	3446	4.2%	0.4	.00	.31	.55
	1–2	1.8%	167	1557	10.7%	0.6	.00	.40	.76
	0	95.9%	9022	51720	17.4%	1.0	ref.	ref.	ref.
**Happiness**	9	0.1%	14	7287	0.2%	0.0	.00	.01	.04
	7–8	0.2%	21	1968	1.1%	0.1	.00	.04	.13
	3–6	1.0%	97	1315	7.4%	0.4	.00	.25	.56
	1–2	2.6%	240	1241	19.3%	1.1	.47	.81	1.56
	0	96.0%	9034	54183	16.7%	1.0	ref.	ref.	ref.
**Sadness**	9	0.4%	40	3004	1.3%	0.2	.00	.11	.28
	7–8	1.2%	116	2819	4.1%	0.5	.00	.31	.69
	3–6	4.9%	464	3602	12.9%	0.5	.00	.39	.68
	1–2	6.0%	568	1803	31.5%	0.8	.14	.59	1.08
	0	87.4%	8218	54766	15.0%	1.0	ref.	ref.	ref.
**Neutral (n=9406)**	9	58.1%	5465	5802	94.2%	321.1	.00	233.97	440.71
	7–8	23.5%	2212	2640	83.8%	115.0	.00	83.69	157.90
	3–6	10.3%	969	1782	54.4%	36.3	.00	27.30	48.21
	1–2	0.9%	86	836	10.3%	8.1	.00	5.10	12.84
	0	7.2%	674	54934	1.2%	1.0	ref.	ref.	ref.
**Fear**	9	0.1%	12	2877	0.4%	0.1	.00	.03	.14
	7–8	0.4%	41	3171	1.3%	0.2	.00	.16	.36
	3–6	1.9%	182	4083	4.5%	0.8	.15	.63	1.07
	1–2	2.3%	214	2002	10.7%	1.0	.82	.75	1.26
	0	95.2%	8957	53861	16.6%	1.0	ref.	ref.	ref.
**Disgust**	9	0.1%	5	4233	0.1%	0.0	.00	.01	.04
	7–8	0.1%	9	2986	0.3%	0.0	.00	.02	.10
	3–6	0.4%	36	3025	1.2%	0.1	.00	.09	.22
	1–2	1.2%	110	1297	8.5%	0.5	.00	.30	.72

	0	98.3%	9246	54453	17.0%	1.0	ref.	ref.	ref.
**Model age**	≥ 46	9.8%	923	6515	14.2%	1.7	.00	1.44	2.00
	26–45	55.4%	5213	36103	14.4%	0.9	.04	.83	1.00
	≤ 25	34.8%	3270	23376	14.0%	1.0	ref.	ref.	ref.
**Model gender**	Female	50.2%	4722	33042	14.3%	1.9	.00	1.68	2.07
	Male	49.8%	4684	32952	14.2%	1.0	ref.	ref.	ref.
**Rater age**	≥ 46	35.7%	3362	23405	14.4%	1.3	.09	.96	1.68
	26–45	50.0%	4702	33054	14.2%	1.0	1.00	.83	1.21
	≤ 25	14.3%	1342	9535	14.1%	1.0	ref.	ref.	ref.
**Rater gender**	Female	70.4%	6622	46600	14.2%	1.3	.03	1.03	1.65
	Male	29.6%	2784	19394	14.4%	1.0	ref.	ref.	ref.

Supplemental Table 6. *Factors associated with images portraying fear. The total falls short of 100% because some of the responses made at each step of the response rating scale for fear were actually made for portrayals of other, unintended emotions, and these figures appear in the corresponding tables for each emotion.*

	**Rating**	**Proportion**	**Ratings made for images portraying fear (n=9211)**	**Ratings made for all images**	**Ratings made for portrayals of fear as a proportion of all ratings**	**Adjusted odds ratio**	***P***	**95 % Wald CI**	
**Anger**	9	1.3%	117	4702	2.5%	0.2	.00	0.1	0.2
	7–8	1.4%	127	3135	4.1%	0.2	.00	0.2	0.3
	3–6	3.6%	335	3575	9.4%	0.6	.00	0.5	0.7
	1–2	2.4%	217	1823	11.9%	1.0	.68	0.8	1.2
	0	91.4%	8415	52759	15.9%	1.0	ref.	ref.	ref.
**Surprise**	9	5.7%	523	5543	9.4%	0.5	.00	0.4	0.6
	7–8	8.5%	781	3728	20.9%	0.8	.10	0.7	1.0
	3–6	15.7%	1449	3446	42.0%	1.8	.00	1.5	2.2
	1–2	5.5%	510	1557	32.8%	2.1	.00	1.7	2.6
	0	64.6%	5948	51720	11.5%	1.0	ref.	ref.	ref.
**Happiness**	9	0.2%	22	7287	0.3%	0.0	.00	0.0	0.0
	7–8	0.3%	30	1968	1.5%	0.1	.00	0.1	0.1
	3–6	1.7%	158	1315	12.0%	0.7	.00	0.6	0.9
	1–2	2.2%	207	1241	16.7%	1.3	.06	1.0	1.6
	0	95.5%	8794	54183	16.2%	1.0	ref.	ref.	ref.
**Sadness**	9	0.3%	30	3004	1.0%	0.0	.00	0.0	0.1
	7–8	0.5%	50	2819	1.8%	0.0	.00	0.0	0.1
	3–6	2.0%	182	3602	5.1%	0.1	.00	0.1	0.2
	1–2	1.8%	163	1803	9.0%	0.4	.00	0.3	0.6
	0	95.4%	8786	54766	16.0%	1.0	ref.	ref.	ref.
**Neutral**	9	0.2%	14	5802	0.2%	0.0	.00	0.0	0.0
	7–8	0.3%	31	2640	1.2%	0.1	.00	0.0	0.1
	3–6	0.7%	65	1782	3.6%	0.3	.00	0.2	0.3
	1–2	0.9%	86	836	10.3%	0.5	.00	0.3	0.8
	0	97.9%	9015	54934	16.4%	1.0	ref.	ref.	ref.
**Fear (n=9211)**	9	27.9%	2572	2877	89.4%	72.0	.00	56.0	92.5
	7–8	27.6%	2540	3171	80.1%	40.4	.00	33.3	48.9
	3–6	23.0%	2115	4083	51.8%	13.7	.00	11.7	16.2
	1–2	3.8%	348	2002	17.4%	4.1	.00	3.3	5.1
	0	17.8%	1636	53861	3.0%	1.0			
**Disgust**	9	0.5%	49	4233	1.2%	0.1	.00	0.0	0.1
	7–8	1.2%	108	2986	3.6%	0.2	.00	0.1	0.2
	3–6	4.6%	424	3025	14.0%	0.6	.00	0.5	0.7

	1–2	2.9%	270	1297	20.8%	1.1	.56	0.9	1.3
	0	90.8%	8360	54453	15.4%	1.0	ref.	ref.	ref.
**Model age**	≥ 46	10.1%	928	6515	14.2%	0.8	.00	0.7	0.8
	26–45	54.9%	5053	36103	14.0%	1.0	.72	1.0	1.1
	≤ 25	35.1%	3230	23376	13.8%	1.0	ref.	ref.	ref.
**Model gender**	Female	50.2%	4621	33042	14.0%	1.0	.78	0.9	1.1
	Male	49.8%	4590	32952	13.9%	1.0	ref.	ref.	ref.
**Rater age**	≥ 46	35.5%	3269	23405	14.0%	1.0	.76	0.8	1.3
	26–45	49.6%	4572	33054	13.8%	1.0	.56	0.8	1.1
	≤ 25	14.9%	1370	9535	14.4%	1.0	ref.	ref.	ref.
**Rater gender**	Female	70.7%	6516	46600	14.0%	1.1	.45	0.9	1.2
	Male	29.3%	2695	19394	13.9%	1.0	ref.	ref.	ref.

Supplemental Table 7. *Factors associated with images portraying disgust. The total falls short of 100% because some of the responses made at each step of the response rating scale for disgust were actually made for portrayals of other, unintended emotions, and these figures appear in the corresponding tables for each emotion.*

	Rating	Proportion	Ratings made for images portraying disgust (n=9325)	Ratings made for all images	Ratings made for portrayals of disgust as a proportion of all ratings	Adjusted odds ratio	*P*	95 % Wald CI	
**Anger**	9	1.0%	91	4702	1.9%	0.2	.00	0.1	0.2
	7–8	1.3%	118	3135	3.8%	0.2	.00	0.2	0.3
	3–6	5.7%	530	3575	14.8%	0.6	.00	0.5	0.7
	1–2	3.7%	346	1823	19.0%	1.1	.27	0.9	1.4
	0	88.4%	8240	52759	15.6%	1.0	ref.	ref.	ref.
**Surprise**	9	0.2%	19	5543	0.3%	0.0	.00	0.0	0.1
	7–8	0.7%	61	3728	1.6%	0.1	.00	0.1	0.2
	3–6	2.4%	226	3446	6.6%	0.3	.00	0.2	0.3
	1–2	2.4%	220	1557	14.1%	0.7	.00	0.5	0.9
	0	94.4%	8799	51720	17.0%	1.0	ref.	ref.	ref.
**Happiness**	9	0.1%	12	7287	0.2%	0.0	.00	0.0	0.0
	7–8	0.2%	14	1968	0.7%	0.1	.00	0.0	0.1
	3–6	0.9%	83	1315	6.3%	0.6	.00	0.4	0.8
	1–2	1.5%	137	1241	11.0%	1.3	.07	1.0	1.7
	0	97.4%	9079	54183	16.8%	1.0	ref.	ref.	ref.
**Sadness**	9	1.0%	97	3004	3.2%	0.3	.00	0.2	0.4
	7–8	1.5%	136	2819	4.8%	0.3	.00	0.2	0.4
	3–6	3.9%	360	3602	10.0%	0.4	.00	0.3	0.5
	1–2	2.6%	246	1803	13.6%	0.7	.00	0.6	0.9
	0	91.0%	8486	54766	15.5%	1.0	ref.	ref.	ref.
**Neutral**	9	0.1%	10	5802	0.2%	0.0	.00	0.0	0.0
	7–8	0.1%	12	2640	0.5%	0.1	.00	0.0	0.1
	3–6	0.4%	34	1782	1.9%	0.2	.00	0.1	0.2
	1–2	1.0%	91	836	10.9%	0.6	.01	0.4	0.9
	0	98.4%	9178	54934	16.7%	1.0	ref.	ref.	ref.
**Fear**	9	0.3%	24	2877	0.8%	0.1	.00	0.0	0.1
	7–8	0.6%	54	3171	1.7%	0.1	.00	0.1	0.1
	3–6	2.5%	233	4083	5.7%	0.2	.00	0.1	0.3
	1–2	2.4%	225	2002	11.2%	0.5	.00	0.4	0.7
	0	94.3%	8789	53861	16.3%	1.0	ref.	ref.	ref.
**Disgust (n=9325)**	9	43.3%	4034	4233	95.3%	299.5	.00	227.3	394.6
	7–8	28.5%	2661	2986	89.1%	163.0	.00	130.5	203.5
	3–6	19.4%	1808	3025	59.8%	50.6	.00	42.2	60.6
	1–2	1.9%	180	1297	13.9%	10.3	.00	7.5	14.2

	0	6.9%	642	54453	1.2%	1.0	ref.	ref.	ref.
**Model age**	≥ 46	9.6%	895	6515	13.7%	1.2	.05	1.0	1.4
	26 – 45	55.3%	5154	36103	14.3%	0.8	.00	0.7	0.8
	≤ 25	35.1%	3276	23376	14.0%	1.0	ref.	ref.	ref.
**Model gender**	Female	50.2%	4677	33042	14.2%	1.0	.86	0.9	1.1
	Male	49.8%	4648	32952	14.1%	1.0	ref.	ref.	ref.
**Rater age**	≥ 46	35.7%	3326	23405	14.2%	1.4	.01	1.1	1.8
	26–45	50.0%	4665	33054	14.1%	1.2	.13	1.0	1.5
	≤ 25	14.3%	1334	9535	14.0%	1.0	ref.	ref.	ref.
**Rater gender**	Female	70.9%	6614	46600	14.2%	0.8	.01	0.7	0.9
	Male	29.1%	2711	19394	14.0%	1.0	ref.	ref.	ref.
